# Empathy Is Moderated by Genetic Background in Mice

**DOI:** 10.1371/journal.pone.0004387

**Published:** 2009-02-11

**Authors:** QiLiang Chen, Jules B. Panksepp, Garet P. Lahvis

**Affiliations:** 1 Undergraduate Program in Clinical Laboratory Science, University of Wisconsin-Madison, Madison, Wisconsin, United States of America; 2 Neuroscience Training Program, University of Wisconsin-Madison, Madison, Wisconsin, United States of America; 3 Department of Behavioral Neuroscience, Oregon Health and Science University, Portland, Oregon, United States of America; University of Parma, Italy

## Abstract

Empathy, as originally defined, refers to an emotional experience that is shared among individuals. When discomfort or alarm is detected in another, a variety of behavioral responses can follow, including greater levels of nurturing, consolation or increased vigilance towards a threat. Moreover, changes in systemic physiology often accompany the recognition of distressed states in others. Employing a mouse model of cue-conditioned fear, we asked whether exposure to conspecific distress influences how a mouse subsequently responds to environmental cues that predict this distress. We found that mice are responsive to environmental cues that predict social distress, that their heart rate changes when distress vocalizations are emitted from conspecifics, and that genetic background substantially influences the magnitude of these responses. Specifically, during a series of pre-exposure sessions, repeated experiences of object mice that were exposed to a tone-shock (CS-UCS) contingency resulted in heart rate deceleration in subjects from the gregarious C57BL/6J (B6) strain, but not in subjects from the less social BALB/cJ (BALB) strain. Following the pre-exposure sessions, subjects were individually presented with the CS-only for 5 consecutive trials followed by 5 consecutive pairings of the CS with the UCS. Pre-exposure to object distress increased the freezing responses of B6 mice, but not BALB mice, on both the CS-only and the CS-UCS trials. These physiological and behavioral responses of B6 mice to social distress parallel features of human empathy. Our paradigm thus has construct and face validity with contemporary views of empathy, and provides unequivocal evidence for a genetic contribution to the expression of empathic behavior.

## Introduction

The detection of discomfort in others can engender a variety of behavioral responses. For instance, distressed conspecifics can prompt vigilance towards a threat, such as an approaching predator, that is not perceived directly [1], [2]. Another example includes the role of infant vocalizations in provoking parental care toward their immediate needs [3], [4]. Moreover, social interactions with a distressed conspecific can activate brain regions that are responsive to a direct experience with threatening stimuli [5].

Detection of distress is also a necessary aspect for social learning. In nature, social learning can confer a substantial benefit, allowing an individual to identify cues that are predictive of a salient situation without directly experiencing the potential threats [6]–[8]. However, social learning often entails the transfer of additional information between individuals, typically related to the performance of operant tasks. Such tasks can include techniques to obtain food [9]–[12] or manoeuvres to avoid predators [13], [14]. Thus, social learning can require a variety of functions, including an ability to perceive distress in others, recognizing when environmental cues co-occur with social distress, and executing a behavioural sequence that minimizes exposure to the indirectly perceived threat (see Ref. [15] for a review). Importantly, distinct biological factors may contribute to each of these abilities. In the present study, we focused specifically on the process by which a mouse acquires fear in relation to a stimulus that is predictive of distress in others, irrespective of its subsequent ability to avoid a threatening situation.

Several definitions of socio-emotional processing, exemplified by the detection and emotional response to discomfort in others, are strikingly similar to perspectives on empathy. Lipps' classical conception of *einfühlung* or ‘feeling into’ [16] was translated to ‘empathy,’ a term that has since been expanded and refined [17]. Contemporary definitions of empathy continue to maintain a primary role for affective reactivity to others; the generation of an affective state more appropriate to the situation of another compared to one's own [18]. In this regard, a recent study demonstrated that mice respond differentially to the level of distress experienced by a nearby conspecific, a finding that was interpreted as a basic form of empathy [19]. However, more developed forms of empathy may be required for social learning. For instance, the experience of conspecific discomfort might influence how a mouse subsequently responds to the environmental cues that predict this distress. Furthermore, although association studies in humans have suggested a genetic contribution to empathic processing [20], it remains unknown whether genetic factors directly influence receptivity to discomfort in others.

We addressed these possibilities in the present study by using a modified cue-conditioned fear paradigm that has been used extensively to elucidate fear circuitry within the mammalian brain [21], [22]. A standard fear conditioning procedure entails presenting an animal with a neutral stimulus, such as a tone (conditioned stimulus, CS), that is forward paired with exposure to an aversive stimulus, such as an electrical shock (unconditioned stimulus, UCS). Upon repeated administration of the paired CS-UCS, a fear response is acquired, which is expressed as freezing in response to presentation of the CS-only. This freezing response is indicative of fear [21] and may be adaptive within natural contexts [23]–[26].

**Figure 1:**
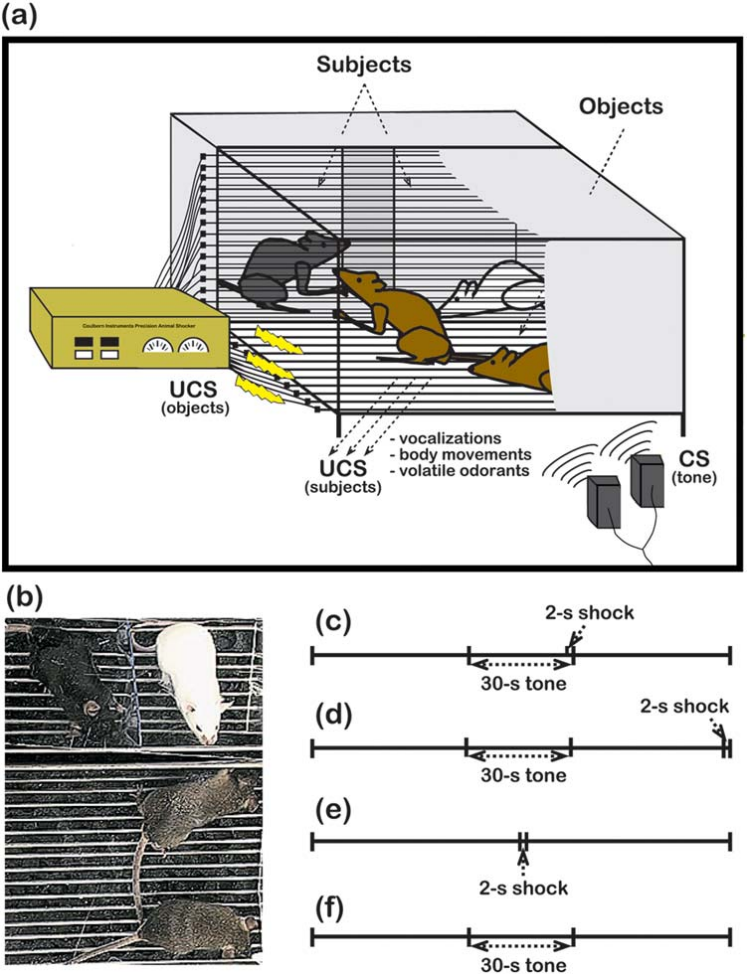
The fear conditioning apparatus and conditioning schedules. (a) Subject mice from the BALB (*white mouse*) and B6 (*dark grey mouse*) strains were separated from 2 novel, F1 object mice (*brown mice*) that received a 2-s electrical shock (UCS _objects_) and/or a 30-s tone (CS) under different conditioning schedules (pre-exposure sessions). Both subjects and objects were exposed to the CS, but only objects directly experienced the UCS. Subjects had access to object distress cues (UCS _subjects_) that were emitted as a consequence of receiving the UCS. (b) Photograph of the fear conditioning apparatus from an overhead perspective. (c–f) Each conditioning schedule is representative of one 120-s trial.

Studies of social learning typically employ the terms ‘*demonstrator*’ and ‘*observer*’ to denote an individual the actively participates in a task versus an individual that learns through social observation, respectively. However, consistent with the empathy literature, we decided to use the terms ‘*object*’ and ‘*subject*’, which bare similarity to the demonstrator–observer nomenclature, but do not imply that individuals perform an operant task. We adopted a protocol that included a series of observation (‘pre-exposure’) sessions in which subjects experienced object mice that were repeatedly exposed to a tone forward paired with a shock (**Figs. 1a–b**). Using this approach, we asked whether the experience of objects undergoing a CS-UCS contingency could subsequently modify subject responses to presentation of the CS-only and to the CS-UCS. Freezing does not require the acquisition of an ability to perform a specific operant task, thereby allowing for more direct comparisons to the large body of data regarding fear learning [21], [22]. Furthermore, we compared mice from the BALB and B6 strains because they express substantial differences in adolescent social motivation [27], [28], which may be associated with the ability to process social information [29].

## Results

### BALB mice acquire freezing responses to a CS-UCS contingency more rapidly than B6 mice

**Figure 2:**
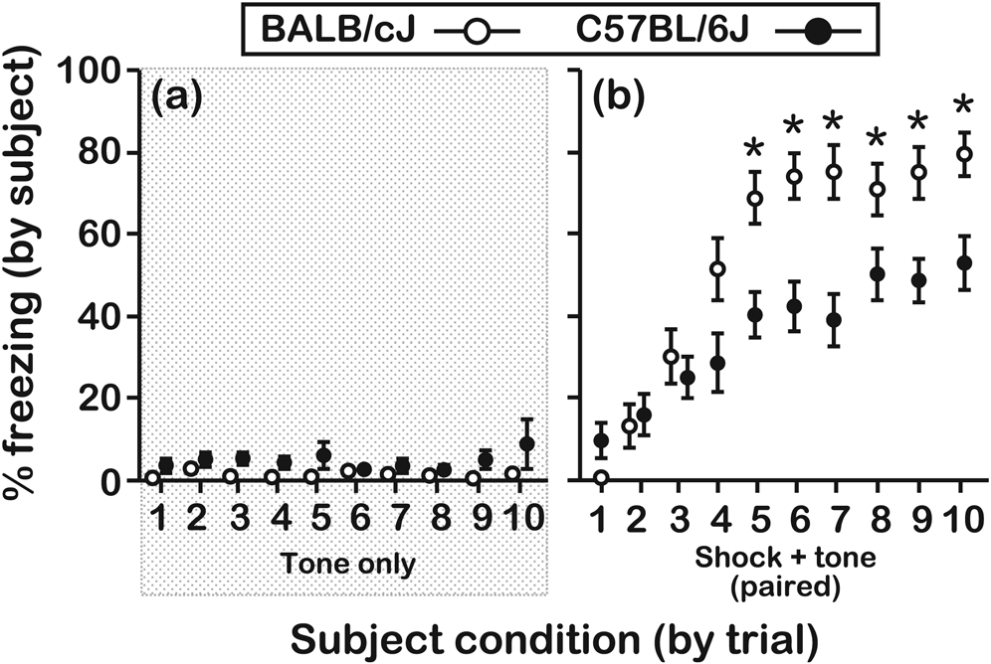
Freezing responses of test mice during direct presentation of the CS-only or the paired CS-UCS. (a) When mice were not pre-exposed to objects or conditioned themselves, there was no strain difference in freezing to the CS (N = 12 mice/genotype; age = 6–7 wks). (b) BALB mice expressed longer freezing responses than B6 mice on trials 5-10 when they had not been pre-exposed to objects, but were directly exposed to the CS-UCS contingency (N = 16 mice/genotype; age = 7–8 wks). Asterisks represent significant differences between BALB and B6 mice as assessed by a Bonferroni step-down procedure on a trial-by-trial basis (*∝* = 0.005 for each trial). All data were scored in duplicate by 2 independent raters (see the figure legend corresponding to Figure 3 for the inter-rate reliability) and are presented as the mean±s.e.m.

To assess how exposure to social distress subsequently influences freezing behavior, it was first necessary to evaluate the freezing responses of BALB and B6 mice to direct presentation of the CS (see **Fig. 1f**) and the paired CS-UCS (see **Fig. 1c**). When test mice were placed individually in a fear-conditioning arena and they were presented with the CS-only (the CS was 30-s tone) for 10 consecutive trials, their freezing responses were minimal and strain-independent (**Fig. 2a**; effect of genotype, F[1], [20] = 1.60, P = 0.22), indicating that presentation of the CS was not salient without conditioning. When test mice were assessed during the first 28 s of a CS that was forward paired with a UCS (the UCS was a 2-s shock) for 10 consecutive trials, test mice from both strains expressed longer freezing responses across successive conditioning trials (**Fig. 2b**). However, with repeated trials a strain difference emerged, as BALB mice froze for longer periods of time than B6 mice on trials 5–10 (genotype×trial interaction, F[9], [20] = 3.53, P = 0.009).

### Pre-exposure of BALB and B6 mice to distressed conspecifics has a differential influence on subsequent freezing behavior to both the CS-only and the paired CS-UCS

We next compared the freezing responses of BALB and B6 subject mice after they had experienced unfamiliar objects receive repeated forward-pairings of the CS with the UCS. Prior to testing, two subjects were placed individually into observation compartments facing the fear-conditioning arena (i.e., the demonstration compartment) that contained objects (**Figs. 1a–b**). After habituation to the demonstration compartment (see **Materials & Methods**), objects received 20 trials (10 trials/session, 1 session/day) of the paired CS-UCS contingency (**Fig. 1c**). During these ‘pre-exposure’ trials, subjects could hear the CS, but did not directly experience the UCS. Approximately 15 min after the second pre-exposure session, freezing responses of subjects were evaluated in the fear-conditioning arena. In this testing phase of the experiment, the freezing behavior of subjects was measured in response to 5 consecutive CS-only presentations followed by 5 consecutive presentations of the CS forward paired with the UCS. Thus, freezing during test trials 1-6 occurred in response to presentation of the tone-only, whereas freezing on test trials 7-10 occurred in the context of direct presentation of the tone-shock contingency. Although the sex of the subject mice was included in all analyses of freezing behavior, no significant effects were found and this independent variable is therefore not considered further.

**Figure 3:**
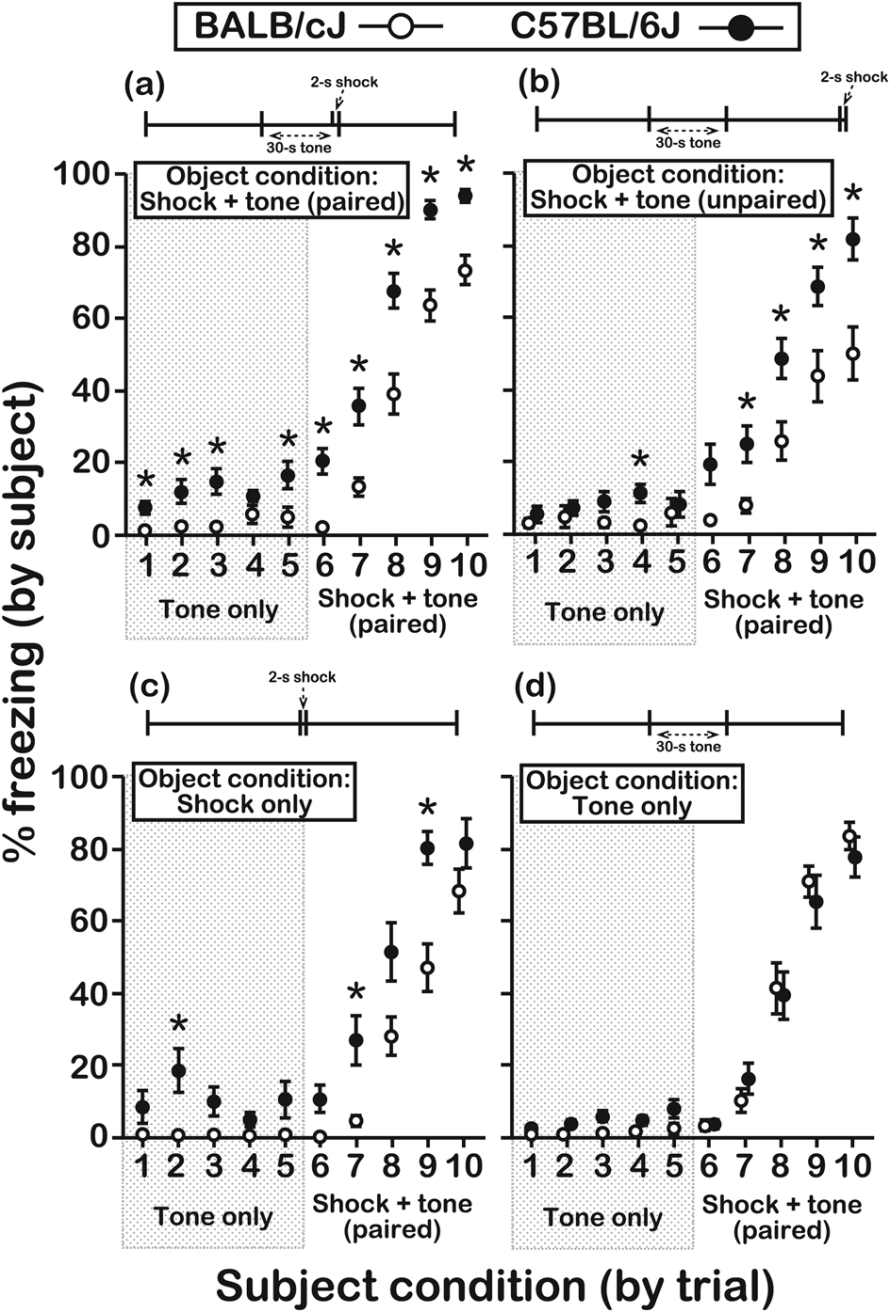
Freezing responses of subject mice to the CS-only and the paired CS-UCS following pre-exposure to object mice under different conditioning schedules. The top of each panel contains a graphical representation of a single conditioning trial of the type that objects received during the pre-exposure sessions. The data in each panel depict the freezing behavior of subjects after pre-exposure sessions in response to the CS before (trials 1-6) and after (trials 7-10) it was forward paired with the UCS. (a) When previously exposed to objects receiving the paired CS-UCS, B6 subjects expressed more freezing than BALB subjects on 9 (of 10) test trials (N = 26 subjects/genotype; age = 5–7 wks). (b) B6 subjects expressed more freezing than BALB subjects on 5 (of 10) test trials when they were previously exposed to objects receiving the unpaired CS-UCS (N = 18 subjects/genotype; age = 5–7 wks). (c) B6 subjects expressed more freezing than BALB subjects on 3 (of 10) test trials when they were previously exposed to objects receiving the UCS-only (N = 14 subjects/genotype; age = 5–6 wks). (d) Freezing responses of BALB and B6 subjects were similar on all test trials when they were previously exposed to objects receiving the CS-only (N = 16 subjects/genotype; age = 5–7 wks). Note that in all cases the UCS was presented during the final 2 s of trial 6, which was not included in the measurements of freezing. Asterisks represent significant differences between BALB and B6 mice as assessed by a Bonferroni step-down procedure on a trial-by-trial basis (*∝* = 0.008 and 0.013 for trials 1-6 and trials 7-10, respectively). All data were scored in duplicate by 2 independent raters and are presented as the mean±s.e.m. Inter-rater reliability, Pearson's correlation, r_p_ = 0.94, d.f. = 1,984.

B6 subjects expressed longer freezing responses than BALB subjects after exposure to objects receiving the CS-UCS contingency (**Fig. 3a**; effect of subject genotype, F[1,124] = 53.8, P<0.0001). CS-only trials (trials 1-6) elicited freezing by B6 subjects for 13.5±1.28% of the time (mean±s.e.m.) compared to 3.0±0.67% of the time for BALB subjects (orthogonal contrast, P<0.001). B6 subjects therefore expressed enhanced freezing in response to the CS even though they had not directly experienced the CS-UCS association themselves. These B6 subjects also exhibited enhanced fear learning (trials 7-10) relative to BALB subjects (orthogonal contrast, P<0.001). After 4 direct experiences with the CS-UCS (trial 10 in **Fig. 3a**), the freezing responses of B6 subjects (93.7±1.66%) were longer than those of experience-matched BALB subjects (73.0±4.06%) and of object-inexperienced B6 mice (42.7±5.89%; trial 5 in **Fig. 2b**).

To further characterize the enhanced freezing responses of B6 subjects, we pre-exposed subject mice of both strains to objects that received different combinations of the CS and UCS (**Figs. 1d–f**; subject genotype×object condition interaction, F[3,124] = 6.24, P = 0.0006). In an initial control experiment, object mice were exposed to the CS and UCS, but the conditioning stimuli were not paired (**Fig. 1d**). Even after pre-exposure to objects experiencing this unpaired CS-UCS, B6 subjects expressed longer freezing responses than BALB subjects (**Fig. 3b**) during several of the CS-only (orthogonal contrast, P = 0.03) and paired CS-UCS (orthogonal contrast, P<0.001) test trials. In a second control experiment, we compared the freezing responses of subjects after they experienced object mice receive the UCS-only (**Fig. 1e**). B6 subjects again expressed longer freezing relative to BALB subjects (**Fig. 3c**) in response to presentation of the CS-only (orthogonal contrast, P = 0.003) and the paired CS-UCS (orthogonal contrast, P<0.001). Consistent with a non-associative influence of UCS presentation on fear responses [30], [31], these findings indicate that B6 freezing is amplified by the experience of object distress regardless of its precise relationship to the CS. Importantly, however, B6 freezing responses after pre-exposure to objects receiving the paired CS-UCS contingency were more robust than after they experienced the other pre-exposure conditions (subject genotype×object condition×trial type interaction, F[9,118] = 2.59, P = 0.009; orthogonal contrast for the paired CS-UCS object condition vs. all other object conditions, P<0.0001). Furthermore, after pre-exposure to object mice receiving the CS-only (**Fig. 1e**), subjects from both strains did not express a freezing response to the CS (orthogonal contrast, P = 0.43) and their responses to the paired CS-UCS were also similar (**Fig. 3d**; orthogonal contrast, P = 0.48).

**Figure 4:**
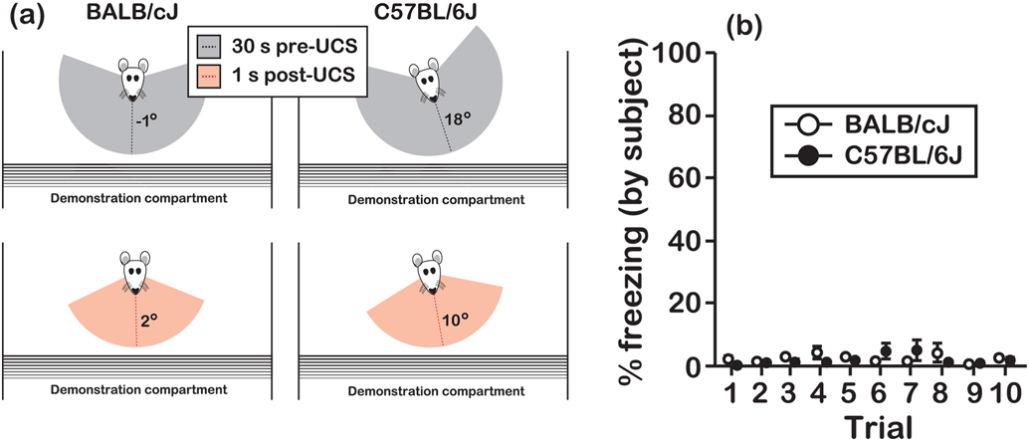
Head orientations and freezing responses of subjects during exposure to object distress. (a) Estimates of subject head orientations were extracted from freeze-frames of video recordings at 2 time-points with respect to UCS presentation to objects (30 s pre-UCS and 1 s post-UCS). The longitudinal axis of the subject's head, running parallel with the sagittal midline, was referenced at 15° increments. A 0° head orientation was defined by the longitudinal axis of the subject's head forming a right angle with the steel dowels that separated the observation and demonstration compartments. Prior to presentation of the UCS to objects, BALB (*left panels*; N = 16 mice; age = 7–8 wks) and B6 (*right panels*; N = 8 mice, age = 7–8 wks) subjects were orientated towards the demonstration compartment (mean head angle = dotted black lines, std. dev. = grey shaded area). Following presentation of the UCS to objects, subject head orientations were more consistently directed at objects (depicted by red dotted lines and pink shading), as indicated by a ≈2-fold reduction in variability of head orientations (Brown-Forsythe ANOVA, P<0.001 for both BALB and B6 subjects). (b) The freezing responses of these subjects were minimal and strain-independent during the 30-s period following presentation of the UCS to objects. Data in panel (b) are presented as the mean±s.e.m.

To determine whether subject mice from either strain responded to objects as they received the UCS, we monitored subjects in another experiment *during* presentation of the UCS to object mice. When objects received the UCS, subjects from both strains oriented towards the demonstration compartment (**Fig. 4a**). However, neither BALB nor B6 subject mice expressed freezing responses immediately after objects received the UCS (**Fig. 4b**; effect of genotype, F[9], [12] = 1.12 P = 0.42). Although we did not quantify this response, it is also worth noting that during many trials of the pre-exposure sessions both BALB and B6 subject mice exhibited a brief eye-closure response that was coincident with object distress.

### Pairing of object ‘distress’ vocalizations with the CS during pre-exposure sessions enhances B6 freezing to subsequent presentation of the CS

**Figure 5:**
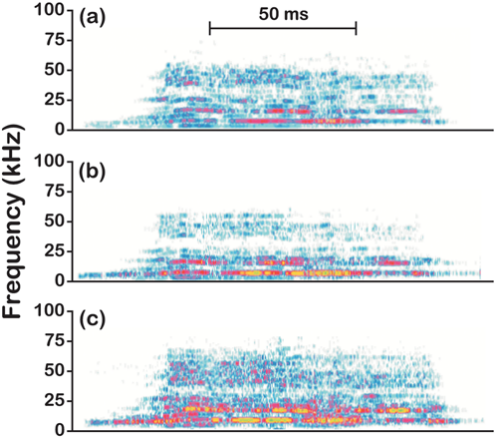
Distress vocalization of an object during UCS presentation. (a) Representative sonogram of an object distress vocalization recorded in real-time without the demonstration compartment enclosed by Plexiglas®. (b) Sonogram of the same vocalization played back through a speaker without the demonstration compartment enclosed. (c) Sonogram of the same vocalization played back through a speaker with the demonstration compartment enclosed. Color-coding indicates the relative decibel level for the call, with blues representing low-intensity energy and red/yellow representing high-intensity energy.

**Figure 6:**
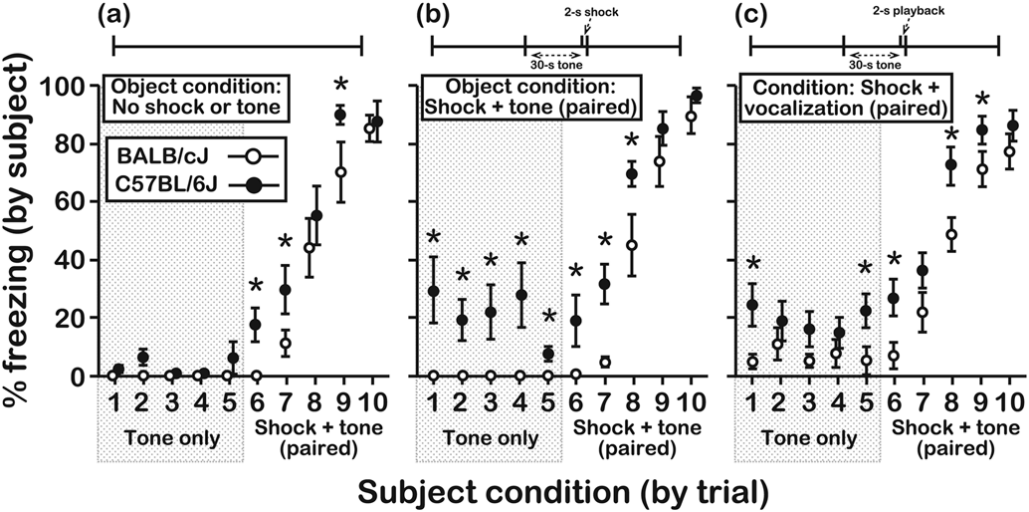
Freezing responses of subjects after pre-exposure to the CS paired with playback of object distress vocalizations. (a) Subjects were exposed to objects during conditioning, but the CS-UCS was not presented to objects (N = 10 subjects/genotype; age = 5–7 wks). (b) Subjects were exposed to objects receiving the paired CS-UCS during conditioning (N = 8 subjects/genotype; age = 5–6 wks). (c) Subjects were not exposed to objects, but received the CS forward paired with the playback of object distress vocalizations during conditioning (N = 16 subjects/genotype; age = 5–7 wks). Asterisks represent a significant (P<0.05) difference between subjects from the BALB and B6 strains. All data were scored in duplicate by 2 independent raters and are presented as the mean±s.e.m. Inter-rater reliability, r_p_ = 0.96, d.f. = 659.

Although visual and somatosensory information could not be transmitted between the objects and subjects during the pre-exposure sessions (see **Materials & Methods**), object distress could have been communicated through auditory or olfactory cues. In fact, object mice consistently emitted audible vocalizations in response to the UCS (**Table 1**). To assess the role of these ‘distress’ vocalizations, we pre-exposed BALB and B6 subjects to recordings of vocalizations of objects receiving the UCS (**Fig. 5**) forward paired with the CS (10 trials/session, 1 session/day). Twenty trials of this contingency was sufficient to reproduce the strain difference in subject freezing, particularly on test trials involving the CS-only (**Fig. 6**; genotype×conditioning context interaction, F[2,56] = 2.11, P = 0.05).

**Table 1:** Descriptive statistics for object distress vocalizations.

Vocalizations/UCS	7.7±0.56 calls
Duration	221.0±170.43 ms
Dominant frequency	19.5±17.55 kHz
Bandwidth	57.0±14.57 kHz

Vocalizations were recorded from 20 different pre-exposure sessions and sonograms were generated for each respective UCS presentation (10/session). Data are presented as the mean±std. dev.

### Heart rate is responsive to both self-distress and social distress and to cues that predict these states

**Figure 7:**
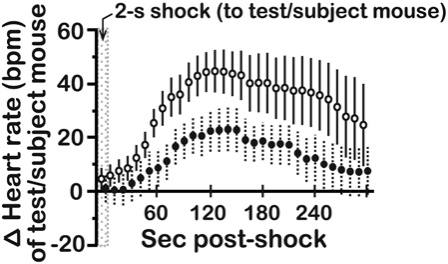
Heart rate changes of mice after a single exposure to the UCS. During habituation to the conditioning apparatus, HR was measured for 5 min following presentation of a single UCS. Mice from the experiments illustrated in Figures 8 and 9 were treated in the same way during the habituation period, and were therefore pooled for graphical presentation and analysis (N = 24 mice/genotype; age = 7–13 wks). Each data point represents a 10-s bin. HR is presented as a change (Δ) from the baseline HR of each mouse (see Table 2). All data presented as the mean±s.e.m.

**Figure 8 pone-0004387-g008:**
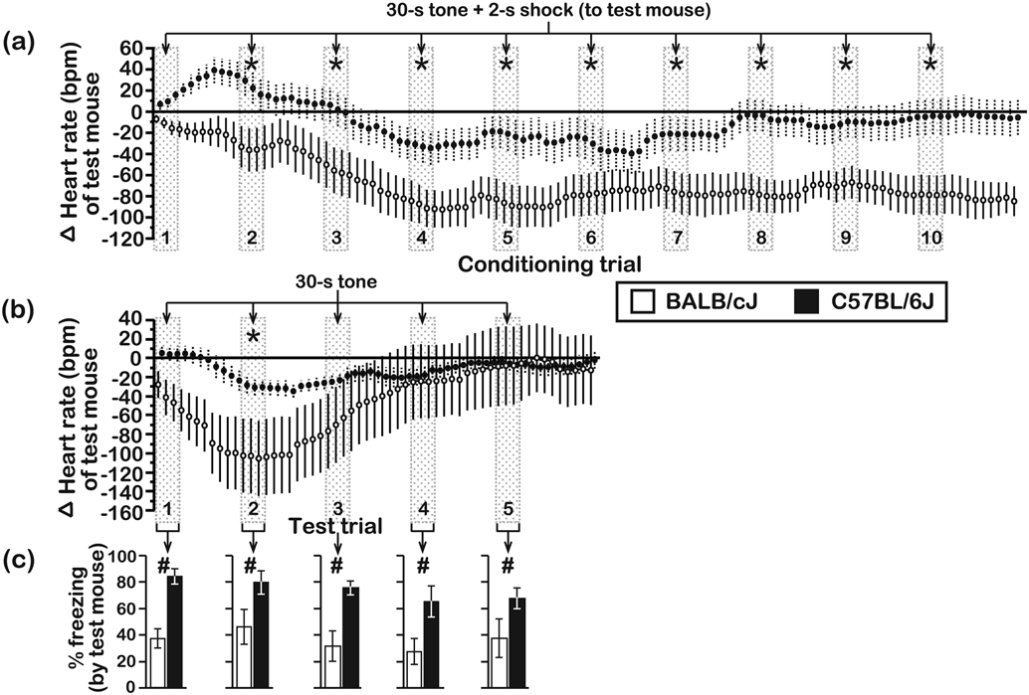
Heart rate changes of mice during direct conditioning with the CS-UCS and testing with the CS-only. HR was measured during (a) the first and second conditioning session in mice that directly received the paired CS-UCS, and during (b) testing, which entailed 5 repeated presentations of the CS-only. HR changes in mice from both strains were similar across the 2 conditioning sessions and therefore pooled for graphical presentation. Each data point represents a 10-s bin. HR is presented as a change (Δ) from the baseline HR of each mouse (see Table 2). (c) Conditioned freezing responses of mice during presentation of the CS-only (N = 9 subjects/genotype; age = 12–13 wks). Asterisks represent significant (P<0.05) HR differences between BALB and B6 mice. Numeric symbols represent significant (P<0.05) differences in freezing between BALB and B6 mice. Behavioral data were scored in duplicate by 2 independent raters and all data are presented as the mean±s.e.m. Inter-rater reliability, r_p_ = 0.90, d.f. = 74.

Physiological parameters, such as heart rate (HR) and skin conductance, provide an additional perspective into the sensitivity of individuals to their own distress and the distress of others [30], [32]. We employed radiotelemetry (see **Materials & Methods**) to monitor the HR of mice either as they experienced the CS-UCS contingency directly or while objects underwent conditioning with the CS-UCS during pre-exposure sessions. The baseline HR of test/subject mice during different phases of these experiments are presented in **Table 2** (maximum percent-changes in HR from baseline ranged from +6.4% to −15.0%). During habituation to the conditioning compartment, a single direct experience of the UCS resulted in elevated output from the heart in both BALB and B6 mice (maximum HR increase±s.e.m.; +40±8 and +23±9 bpm for BALB & B6 mice, respectively), which peaked ≈120 s after the UCS was applied (**Fig. 7**). Repeated presentation of the CS-UCS contingency directly to BALB test mice engendered progressive HR deceleration (**Fig. 8a**, maximum HR decrease±s.e.m.; −78±13 bpm), whereas B6 test mice experienced an initial HR increase, which peaked prior to the second conditioning trial (+32±15 bpm), followed by a HR deceleration that reached a maximum during the sixth conditioning trial (−78±15 bpm). The HR of B6 mice returned to its preconditioning level by the final conditioning trial (**Fig. 8a**). During testing, which entailed 5 successive CS-only trials, HR deceleration in both BALB and B6 test mice (−78±29 and −40±9 bpm for BALB & B6 mice, respectively) reached a maximum during the second test trial before returning to baseline levels by the final test trial (**Fig. 8b**). B6 freezing was longer than BALB freezing on all of the CS-only test trials (**Fig. 8c**), indicating that B6 mice had stronger fear responses than BALB mice upon ‘recall’ of the CS-UCS association.

**Table 2:** Baseline heart rates of test/subject mice during different phases of the conditioning protocol.

	BALB/cJ	C57BL/6J
Habituation (Fig. 7; test/subject mice)	686±11 bpm	754±8 bpm
Conditioning (Fig. 8; test mice)	700±13 bpm	745±16 bpm
Testing (Fig. 8; test mice)	705±24 bpm	776±15 bpm
Conditioning (Fig. 9; subject mice)	671±14 bpm	761±5 bpm
Testing (Fig. 9; subject mice)	674±22 bpm	760±16 bpm

Baseline heart rates of test/subject mice were calculated from a 90-s period that occurred immediately before the beginning of each habituation, conditioning/pre-exposure and testing period. Each of these periods began 20 min after a mouse was placed in the conditioning apparatus. There were no within-strain differences in baseline heart rate across different phases of the conditioning protocol. A previously described strain difference in baseline heart rate [42] was found during all phases of the experiments (P<0.01). Data are presented as the mean±s.e.m.

**Figure 9:**
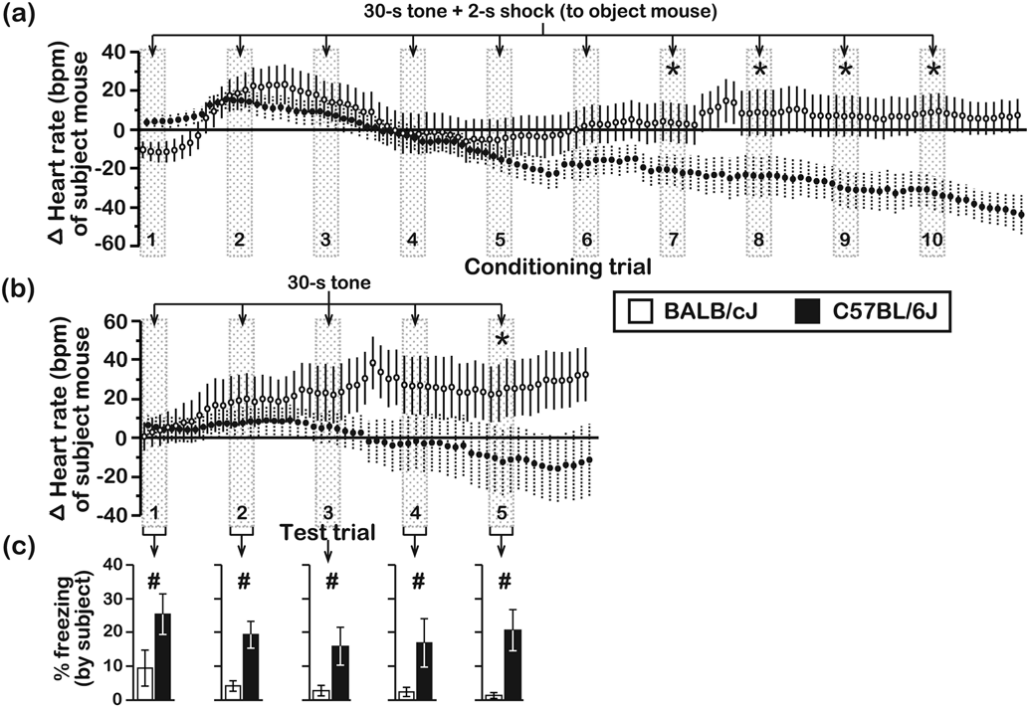
Heart rate changes of subjects during pre-exposure to objects that were conditioned with the CS-UCS and during testing with the CS-only. HR was measured during (a) the first and second conditioning session in subjects that experienced objects receiving the paired CS-UCS, and during (b) testing, which entailed 5 repeated presentations of the CS-only. HR changes in subjects from both strains were similar across the 2 conditioning sessions and therefore pooled for graphical presentation. Each data point represents a 10-s bin. HR is presented as a change (Δ) from the baseline HR of each mouse (see Table 2). (c) Conditioned freezing responses of subjects during presentation of the CS-only (N = 15 subjects/genotype; age = 7–9 wks). Asterisks represent significant (P<0.05) HR differences between BALB and B6 subjects. Numeric symbols represent significant (P<0.05) differences in freezing between BALB and B6 subjects. Behavioral data were scored in duplicate by 2 independent raters and all data are presented as the mean±s.e.m.. Inter-rater reliability, r_p_ = 0.94, d.f. = 143.

In another set of experiments, we monitored the HR of BALB and B6 subject mice as they experienced objects being exposed to the CS-UCS. During the early portion of these pre-exposure sessions, the HR of BALB and B6 subjects accelerated (**Fig. 9a**; +35±8 bpm and +12±4 bpm for BALB & B6 subjects, respectively). However, the HR of BALB subjects returned to baseline levels by the final conditioning trial, whereas the HR of B6 subjects decelerated (−48±10 bpm) below its starting level. During the CS-only trials of the testing session, the HR of BALB and B6 subjects gradually diverged (**Fig. 9b**), and a strain-dependent difference was evident by the fifth CS-only trial. Importantly, the strain-dependent difference in freezing to presentation of the CS was reproduced on all test trials (**Fig. 9c**). It is also noteworthy that, irrespective of the conditioning context (i.e., direct conditioning of test mice or pre-exposure of subject mice to objects receiving conditioning), HR deceleration during conditioning was always associated with pronounced freezing responses during the subsequent CS-only test trials.

### Social investigation and social reward differ between BALB and B6 mice

**Figure 10:**
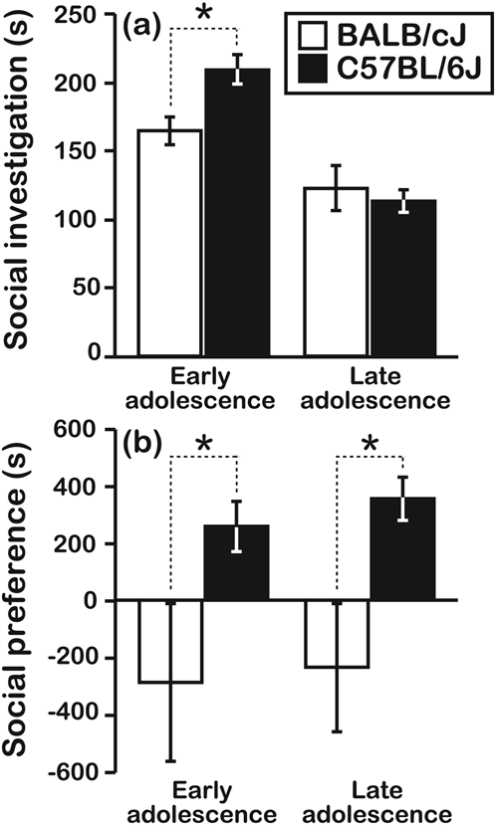
Strain-dependent development of sociability in adolescent mice. (a) Following 24 h of social isolation, early-adolescent (age = ≈4 wks) B6 test mice expressed more social investigation (SI) towards a novel, opposite-sex F1 mouse compared to age-matched BALB mice. There was no difference in SI among late-adolescent mice (age = 7–8 wks) from the BALB and B6 strains (N = 14 mice/genotype/age). The numeric symbol represents a significant (P = 0.01), planned orthogonal contrast between BALB and B6 mice. (b) Following 8 days of conditioning during early adolescence or late adolescence, B6 mice expressed a social conditioned place preference (SCPP), whereas BALB mice expressed social indifference at both time points (N = 14 mice/genotype/age). SCPP scores were calculated by subtracting the time a mouse spent in an isolation-paired environment from the duration it spent in a socially paired environment (see Materials & Methods). The asterisks represent significant (P<0.05), planned orthogonal contrasts between BALB and B6 mice. All data are presented as the mean±s.e.m.

The finding that B6 freezing was sensitive to conspecific distress, but BALB freezing was not, is remarkable because mice from these strains also differ in adolescent social motivation [27], [28]. Using a social investigation (SI) paradigm, we found that the magnitude of SI by early-adolescent B6 mice was greater than that of age-matched BALB mice towards a novel F1 conspecific (similar to the object mice used during the pre-exposure sessions of the earlier experiments), although this strain difference was not maintained through late adolescence (**Fig. 10a**; genotype×age interaction, F[2,48] = 6.50, P = 0.01). Furthermore, B6 mice expressed a socially conditioned place preference (SCPP) throughout early and late adolescence (**Fig. 10b**). By contrast, BALB mice were indifferent to social opportunities at both stages of adolescent development (effect of genotype, F[1,48] = 8.77, P = 0.005; genotype×age interaction, F[2,48] = 0.01, P = 0.91).

### Social isolation suppresses the influence of object distress on B6 freezing

**Figure 11:**
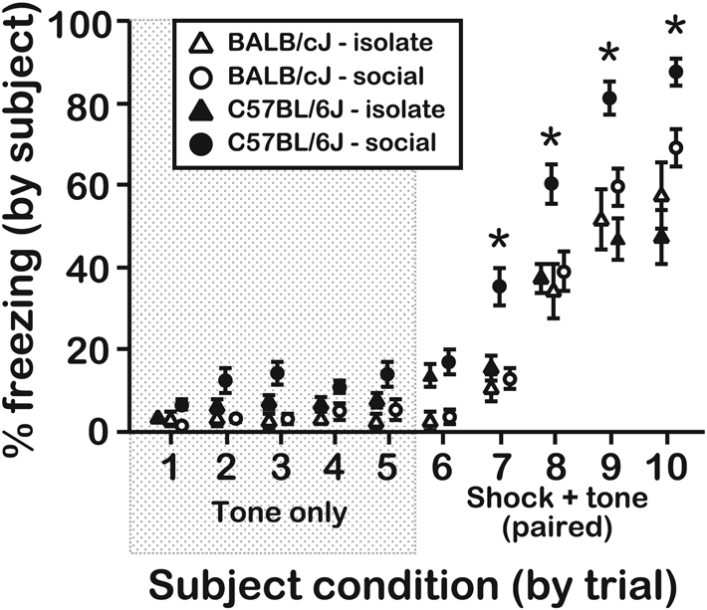
Freezing responses of socially and isolate-housed subjects pre-exposed to objects receiving the CS-UCS contingency. Subjects were either isolated or remained in a social housing context outside of their observations of objects receiving the paired CS-UCS during the pre-exposure session. Since freezing responses of socially housed subjects in this experiment (N = 10 subjects/genotype; age = 6–8 wks) did not differ (P = 0.20) from responses of mice that were evaluated in the experiment presented in Figure 3a, the data were pooled for graphical presentation and statistical analysis. Social isolation did not alter the freezing responses of BALB subjects, but it depressed fear acquisition to presentation of the paired CS-UCS in B6 subjects (N = 20 subjects/genotype; age = 7–8 wks). Socially housed B6 subjects expressed longer freezing responses than all other groups on trials 7-10 (P = 0.006). Asterisks represent significant differences between socially housed B6 subjects and all other groups as assessed by a Bonferroni step-down procedure on a trial-by-trial basis (*∝* = 0.013 for trials 7-10). All data were scored in duplicate by 2 independent raters and are presented as the mean±s.e.m. Inter-rater reliability (not including pooled data from Fig. 3a), r_p_ = 0.94, d.f. = 547.

Our findings suggest that B6 mice might be more sensitive to manipulations of their social environment than BALB mice. To test this idea, we conducted an experiment in which subjects were isolated 24 h prior to being exposed to objects (to increase social motivation during the pre-exposure sessions; see Ref. [33]). The freezing responses of B6 subjects during testing were sensitive to social isolation, particularly during CS-UCS test trials, whereas BALB freezing did not change as a function of social isolation (**Fig. 11**; subject genotype×housing condition interaction, F[1,96] = 7.60, P = 0.007). Thus, the enhanced B6 freezing response following pre-exposure to distressed conspecifics was sensitive to the social housing conditions that preceded testing.

## Discussion

During the present studies, a learning process was engaged as subject mice experienced conspecifics in distress. Following pre-exposure to objects that underwent CS-UCS conditioning, B6 mice expressed freezing responses to the CS-only (test trials 1-6), whereas B6 mice that were pre-exposed to non-distressed objects did not respond appreciably to presentation of the CS. Thus, B6 subjects acquired a classical conditioning (Pavlovian) association, which engendered a freezing response that was dependent upon the previous experience of distress in nearby conspecifics. This social learning process was not evident in BALB mice; they did not freeze in response to the CS-only after pre-exposure to distressed objects. CS-induced freezing after Pavlovian conditioning is thought to reflect a state of fear [21], [22]. The augmented freezing response of B6 subject mice on test trials 1-6 therefore represents the social transfer of fear from objects to subject.

Following pre-exposure to distressed object mice, B6 subjects also expressed enhanced acquisition of conditioned fear (relative to other groups) during the test trials in which they were directly exposed to the paired CS-UCS contingency (test trials 7-10). This enhanced B6 freezing response during CS-UCS conditioning may reflect a persistence of the effect that was evident on test trials 1-6. Alternatively, enhanced B6 freezing during the CS-only and CS-UCS test trials may be due to an independent influence. For example, while the longer freezing responses of B6 mice on trials 1-6 is mediated by the passive transfer of fear from objects to subject, enhanced B6 freezing on trials 7-10, by contrast, could reflect the combined influence of this effect and individual learning, or it could be attributable to a distinct process, such as social facilitation of individual learning. Importantly, we also do not yet know whether the enhanced B6 freezing response occurs specifically to presentation of the CS, or whether it also depends on concurrent recognition of it's own spatial position relative to the appropriate (contextual) environment.

The enhanced B6 freezing response (relative to BALB mice) was not directly related to a difference in the ability to learn associations or to orient to distressed objects. These observations indicate that there may be strain-dependent differences in the emotional responses of mice to social distress. In this regard, a basic form of empathy (i.e., emotional contagion) was recently identified in mice [19]. In these experiments, subject writhing behavior in response to pain was modulated by the degree of concurrent writhing generated by nearby objects [19], analogous perhaps to infectious crying among babies [34]. While our results are similar to the demonstration of emotional contagion in mice, they also bare important differences. First, the subject mice in the present experiments expressed minimal freezing behavior immediately after the induction of object distress. Moreover, freezing responses of the subject mice tested here were measured when objects (and their associated distress cues) were not present in the testing arena. Thus, the augmented freezing behavior of B6 subjects in the present experiments is not directly attributable to a difference in emotional contagion. Lipps (1903) characterized empathy as a process by which “the perception of an emotional gesture in another [*object*] directly activates the same emotion in the perceiver [*subject*], without any intervening labelling, associative or cognitive perspective-taking processes” (passage quoted from ref. [17], pp. 2; bracketed italics added by us). Using this definition, empathy may be widespread among mammalian species [34]. In this regard, the enhanced responses of B6 mice to CS presentation, without any previous experience of self-distress in relation to the CS, may result from an association between the CS and the embodiment of others' distress.

The capacity to learn associations between emotions in others and the cues that predict these states may be a developmental substrate for the emergence of more sophisticated forms of empathy [29]. The ability to rapidly learn an associative relationship through the experience of others' distress has been demonstrated by studies in which mice learn to avoid predatory flies [13], [14], [35]–[37]. In these studies, mice displayed conditioned hypoalgesia and active burying responses upon exposure to non-biting (altered) flies if they had previously observed a demonstrator self-burying 24 hours earlier to avoid biting flies. This burying response is particularly interesting because it was not evident in naïve mice that were exposed to altered flies [13] nor did observer mice bury themselves when demonstrators were concurrently exposed to biting flies [14]. Thus, while the increased pain tolerance of observer mice in this paradigm was dependent on the experience of demonstrators being bitten (presumably mediated through several senses), their learned active avoidance (burying) response was additionally contingent upon visual detection of a demonstrator actively burying itself in bedding to escape the biting flies [13], [14]. Our findings demonstrate that mice can acquire salient, emotional information from conspecifics even when they cannot observe the object's behavioral response to the UCS. Importantly, empathy does not require performance of a specific behavioral response. Thus, while B6 subjects expressed enhanced freezing responses following pre-exposure to object distress, we cannot rule out the possibility that BALB mice may also express empathy. Empathy in BALB mice may be manifest through behavioral responses other than CS-induced freezing, may be manifest only at a physiological level, or may occur under different conditions, such as experiencing distress in objects that are more familiar (e.g., see Refs. [19], [37]).

The human empathy literature consistently reports positive correlations between measures of empathic responsiveness and personality-trait indices of sociability [29]. It is thus interesting that B6 mice exhibit high levels of sociability, social motivation and social reward relative to BALB mice. Previous studies have demonstrated that adolescent B6 mice express pronounced levels of social investigation [27], prefer areas where conspecifics are located [38], [39] and return to environments where social experiences have occurred [28]. By contrast, BALB mice have been found to be much less responsive, or even averse [39] to similar social opportunities. Although general measures of sociability have been associated with empathy in humans, an interesting possibility arising from these studies is that individuals who are more likely to derive reward from a social interaction may also be more prone to embody the emotional responses of others.

The HR changes that occurred in BALB and B6 subjects during the pre-exposure sessions to distressed objects are also consistent with features of human empathy. During the pre-exposure sessions, both BALB and B6 subjects initially oriented towards objects receiving the UCS and exhibited an initial increase in HR, but they did not freeze. The HR of B6 subjects then decelerated during repeated experiences of object distress. This finding is similar to patterns that have been described in children who self-report empathic feelings in relation to the experience of distress in others [40]. Importantly, although there was a general association between test/subject mouse HR deceleration during self/object conditioning and subsequent freezing to the CS-only (B6 subjects, and BALB and B6 test mice froze, while BALB subjects did not), the magnitude and timing of the HR deceleration was not precisely related to the duration of the subsequent freezing response. Additional studies are required to determine the sympathetic versus parasympathetic contribution to HR deceleration during the experience of self-distress and others' distress, and whether this HR deceleration is a necessary feature of fear learning.

Overall, our paradigm bears face and construct validity with contemporary views of empathy [16]–[18], [20], [41]. Animal models that deconstruct empathy into a collection of endophenotypes can facilitate discovery of its underlying biological substrates. Moreover, employing such an approach in mouse genetics could be highly relevant to identifying susceptibility factors for psychosocial disorders, such as autism.

## Materials and Methods

### Ethics statement

All animal care and experimental protocols were conducted in accordance with the regulations of the institutional care and use committee at the University of Wisconsin-Madison and the NIH *Guide for the Care and Use of Laboratory Animals* (ISBN 0-309-05377-3). Personnel from our own laboratory carried out all aspects of the mouse husbandry under strict guidelines to insure careful and consistent handling of the mice.

### Mouse husbandry and housing

Mice from the BALB/cJ (BALB) and C57BL/6J (B6) strains were purchased from Jackson Laboratories (Bar Harbor, ME) and bred within our colony at the University of Wisconsin (Madison, WI) under tightly controlled temperature (21±1°C), humidity (50–60%) and lighting (14∶10 h light/dark, dark period from 1130–2130) conditions. New mice were routinely introduced to the breeding colony and brother-sister matings were not conducted. Mice were maintained under specific-pathogen free conditions and housed in standard polypropylene cages (290×180×130 mm) that contained 1/8″ grain-size corncob bedding (The Andersons) and nesting material (Ancare Corp.) with *ad libitum* access to food (Teklad Rodent Diet, Harlan) and water. Pregnant females were isolated 10–15 days post-coitus and pups were weaned on postnatal day (PD) 20–21 (day of birth = PD 0). Subjects were weaned into mixed-sex social groups containing 4 age-matched cage mates, with 1 mouse/sex from each strain. Subject mice were housed in these mixed-strain social groups to give them experience with mice of another genotype and to reduce the influences of BALB- and/or B6-specific group structure on individual behavior. Objects were derived from reciprocal F1 crosses between BALB and B6 mice and were housed in mixed-sex social groups of 3–5 individuals/cage.

### Fear-conditioning apparatus

The fear-conditioning arena, which was fabricated from ABS plastic and Plexiglas^®^, contained a ‘demonstration’ compartment (130×165×150 mm) and two adjacent ‘observation’ compartments (65×82.5×75 mm per observation compartment). The floor and one wall of the demonstration compartment were lined with a shock grid composed of stainless steel dowels (3.2 mm diameter) spaced 9.6 mm on center. A wall, consisting of two sets of horizontal steel dowels extending vertically 75 mm from the floor, separated the observation and demonstration compartments. This wall allowed subjects to smell and hear objects, but eliminated direct contact with the objects or the UCS (see **Figs. 1a–b**). Scrambled current was provided to the dowels lining the demonstration compartment, but not to those lining the observation compartments. Within the observation compartments, floors were lined with inactive stainless steel dowels and a plastic wall separated each observation compartment. The CS was a 30-s tone (1 kHz, 85 dB) delivered through Dell^®^ computer speakers. The UCS was a scrambled, 2-s electrical shock (0.5 mA) delivered by a Coulbourn^®^ Precision Animal Shocker (Model H13–15).

### Fear-conditioning procedures

All behavioral experiments were conducted under dim red illumination (30–40 lx) during the dark phase of the light-dark cycle (1300–2100). Cages containing the subjects and objects were transported from the mouse colony ≈5 m across a dimly lit intervening room to a procedure room >30 min prior to the beginning of all phases of the conditioning protocol (see below). For most experiments, subject mice lived together for 2–4 weeks prior to conditioning. Individual subjects were habituated to the fear-conditioning arena for a 5-min period of free-exploration followed by presentation of a single 2-s UCS (see Ref. [17]) prior to being returned singly into a clean cage. After completion of the habituation sessions for 2 subject mice, 2 age-matched objects were randomly selected from a cage and placed together in the demonstration compartment. We decided to use 2 objects instead of one to enhance the influence of social distress. Then, the 2 subjects, which included same-sex individuals from different strains, were placed separately into the observation compartments. Home cages that contained the remaining subjects and objects were removed from the procedure room while their cage mates were pre-exposed to object mice receiving conditioning.

Object mice received 10 consecutive 120-s trials under one of several conditioning schedules (see **Figs. 1c–f**), while subjects remained in adjacent observation compartments, but did not receive the UCS. Following the first pre-exposure session, subjects and objects were re-grouped within their respective home cage. The next day subjects received another pre-exposure session as objects underwent another series of 10 conditioning trials. Following the second pre-exposure session, each subject was placed singly into a clean cage and one cage was transported to a holding room adjacent to the procedure room. Subjects were tested ≈15 min after the second pre-exposure session in a randomized order. Testing entailed placing an individual subject in the fear conditioning arena and exposing it to five 120-s, CS-only trials (trials 1-5) followed by five 120-s trials with the CS-UCS presented in a paired fashion (trials 6-10). All testing sessions were videotaped with a 3CCD digital video camera (GL2, Canon Inc., Japan). Recordings were transferred via a firewire cable directly to a Dell™ Pentium® IV desktop computer and stored for subsequent analysis.

The small size of the observation compartments that were used in these experiments made it difficult to accurately measure the freezing behavior of subject mice. Thus, for the experiments presented in **Figure 4**, freezing responses of subjects to object distress were evaluated within an enlarged observation compartment fitted to the size of the demonstration compartment (130×165×150 mm). Subjects were habituated to this observation compartment for 10 min (without objects present) on the day prior to testing. On the day of testing, two F1 objects were placed in the demonstration compartment and 1 subject was placed in the observation compartment. Object mice received a 2-s shock every 120 s.

For experiments that involved social isolation (see **Fig. 11**), subjects were placed individually into a clean home cage 24 h prior to habituation to the demonstration compartment. Socially housed controls were placed as a group into a clean home cage 24 h before their habituation session. The mice used in this experiment were previously evaluated for SI and SCPP (see **Fig. 10** and below), and these individuals were the only mice that were assessed behaviorally more than once. The conditioning apparatus was always cleaned thoroughly with 70% ethanol before introducing new subjects/objects to the conditioning apparatus.

### Vocalization recording

Mouse vocalizations were recorded during 20 different pre-exposure sessions. An ultrasound microphone (UltraSoundGate model CM16, Avisoft Bioacoustics) was inserted through a 30-mm diameter opening (75 mm above the shock grid on center) in the Plexiglas® wall of the demonstration compartment enclosure. Vocalizations were collected with an UltraSoundGate 116 acquisition system and the Avisoft-Recorder v.2.97 (Avisoft Bioacoustics), and stored as wav files for subsequent analysis. Sonograms were generated for each conditioning session and evaluated (SASLab Pro v.4.39, Avisoft Bioacoustics) for the following vocalization parameters: total number (audible and ultrasonic), duration, mean dominant frequency, and bandwidth.

### Vocalization-playback experiments

Recordings of object vocalizations during UCS presentation were reproduced for subjects through an ultrasound-capable speaker (UltraSoundGate ScanSpeak, Avisoft Bioacoustics) situated in the demonstration compartment. To obtain recordings of vocalizations without enclosure-induced distortions, 2 F1 mice (1/sex) were tethered to an open shock grid and exposed to the UCS (see **Fig. 5**). Fifteen representative recordings were sampled from this pair of mice. During the pre-exposure sessions, subjects were exposed to 10 consecutive CS-vocalization forward-pairings per session (randomly selected vocalizations were paired with the CS without substitution during each pre-exposure session). Vocalizations were played back at 85–92 dB. Thus, subjects were exposed to the distress vocalizations of object mice, but were not exposed to objects directly. Following the second pre-exposure session, subjects were assessed in a manner consistent with the previously described testing protocol. The freezing responses of subject mice from this experimental group were compared to responses of groups of additional subjects that either were exposed to objects receiving no conditioning or paired CS-UCS presentation during conditioning.

### Heart rate monitoring

A radiotelemetric transponder (G2-HR E-Mitter®) was surgically implanted into the peritoneum under isoflurane anesthesia according to manufacturer's instructions (Respironics Inc.). Positive and negative leads ran subcutaneously rostral from the transponder, and were affixed to the pectoral/chest muscles with stainless steel sutures (34-gauge). Surgical wounds were closed with polydioxanone sutures (5-0; PDS II, Ethicon Inc.) and cyanoacrylate (Krazy® Glue). Mice were treated postoperatively with a topical 4% (w/w) lidocaine cream (Ferndale Laboratories Inc.) and 0.25% (w/v) bupivicaine HCl (0.1 ml s.c.) for pain management. Mice were allowed to recover for 7–14 days following the surgery. At the beginning of the experiments, the weight of each mouse was either equal to or greater than their respective preoperative weight.

#### Habituation of test/subject mice

Individual mice were placed within the demonstration compartment, above a telemetry receiver, and left undisturbed for 20 min. A single 2-s UCS was then delivered to the subject mouse and HR was monitored for 5 min post-UCS.

#### Conditioning/pre-exposure sessions

For the experiment presented in **Figure 8**, individual test mice were placed in the ‘demonstration’ compartment, acclimated for 20 min and then conditioned directly with the paired CS-UCS. For the experiment presented in **Figure 9**, an individual subject was placed within a randomly selected observation compartment and acclimated for 20 min on the telemetry receiver while 2 novel, age-matched objects (one F1 mouse/sex) were present within the demonstration compartment. Object mice were then exposed to 10 consecutive CS-UCS forward-pairings separated by 90-s intervals. Unlike other experiments where 2 subjects were concurrently pre-exposed to object distress, HR was measured from a single subject per pre-exposure session because the telemetry receiver was only capable of monitoring the signals from one transponder at a time.

#### Testing

A single test/subject mouse was placed in the demonstration compartment and left undisturbed for 20 min on the telemetry receiver. Each mouse was then exposed to 5 consecutive presentations of the CS-only spaced by 90-s intervals.

Signals from the transponders were sampled from mice at 10-s intervals throughout all phases of the conditioning protocol. Per the manufacturer's recommendation, raw signals were subjected to the boxcar (buffer size = 120) and exponential (exp. = 4) filters prior to data analysis (VitalView v.4.1, Respironics Inc.) Following completion of the 20-min period that preceded each phase of the experiments, baseline HR for each subject was derived from the first 90 s of HR data. Data in **Figures 7** **–** [Fig pone-0004387-g008] **9** are presented as deviations from this baseline measurement.

### Behavioral measurements of conditioned fear

Freezing behavior was operationally defined as the complete absence of movement, other than normal respiratory activity. The duration of freezing behavior was recorded over the period of testing when the CS was administered. Two independent observers made the behavioral measurements using computer-assisted software (ButtonBox v.5.1, Behavioral Research Solutions). Inter-rater reliabilities were ≥0.90, with no significant variation between different experiments (the inter-rater reliability of each experiment is presented in the legend of its respective figure). Mean values from the duplicate measurements were used for all data analysis and graphical presentations. Percent-freezing measurements were generated by dividing the raw freezing data either by 30 s (for trials 1-5) or 28 s (for trials 6-10), respectively. Note that freezing on trial 6 was in response to presentation of the CS-only because the UCS was not applied until the final 2 s of the trial.

### Social conditioned place preference procedure

Juvenile mice (PD 20/21) were weaned into a mixed-sex, mixed-strain social group of 4 mice (1 mouse/sex/strain) and housed together for 24 h on corncob bedding with nesting material. On the next day, groups of mice were re-housed as a social group in a novel, home cage environment that contained either aspen shavings (Nepco, Northeastern Products Corp.), two threaded schedule 40 1″ PVC couplers and nesting material or paper bedding (Cellu-Dri™ Soft™, Shepherd Specialty Papers), two unthreaded schedule 40 1″ PVC couplers and nesting material. Mice were first housed as social groups in one of the environments for 24 h and then each mouse was socially isolated in the alternative environment for the next 24 h. Mice were alternated between social- and isolate-housing for a total of 8 days. On the final 2 days of conditioning, mice were habituated individually for 20 min to a 3-compartment testing arena (300×150×150 mm/compartment) that was fabricated from black ABS plastic prior to being placed in the new conditioning environment. No conditioning cues were present during the habituation sessions and mice could freely explore all 3 compartments of the arena by passing through small openings (50×50 mm) in the walls that separated each compartment. Habituations and testing took place during the dark phase of the circadian cycle (1600–2000) under dim red illumination (30–40 lx) and cages were transported to the procedure room >30 min prior to habituation and testing sessions. Mice were always tested after 24 h of social isolation. For testing, individual mice were placed in the central compartment of the conditioning arena, which contained no conditioning cues. Each peripheral compartment of the arena contained one of the 2 conditioning environments (i.e., bedding and PVC tubes). The arena was covered with a transparent sheet of Plexiglas® and mice (early adolescence; postnatal [PD] 29–30) were videotaped for 30 min as they moved throughout the arena. Late-adolescent mice (tested on PD 48–53) remained together as a social group until conditioning, and then the husbandry schedule and conditioning procedure used for early-adolescent mice was followed. Socially conditioned place preference (SCPP) scores were calculated by subtracting the time each mouse spent in the isolation-paired environment from the time spent in the socially paired environment. The pairing of the aspen and paper environments with social and isolate housing was pseudo-randomized and counter-balanced across and within the 2 age groups. Following SCPP testing, mice were re-grouped with their former cage mates into a clean cage that contained corncob bedding and nesting material.

### Social investigation tests

Twenty-four to 48 h after SCPP testing, test mice were placed alone in cages with fresh corncob bedding and remained in social isolation for 24 h prior to testing. Cages were transported to the procedure room >30 min prior to testing and testing took place during the dark phase of the circadian cycle (1600–2000) under dim red illumination. Cage tops were removed and replaced with a transparent piece of Plexiglas® 5–10 min before testing began. Test mice were presented with a novel, age-matched F1 mouse of the opposite sex and social interactions were videotaped for 5 min. Measurements of SI entailed the total duration that the test mouse directed pro-social behaviors at the stimulus mouse, as described in more detail elsewhere (see Ref. [27]). Early-adolescent mice were tested on PD 31–32 and late-adolescent mice were tested on PD 52–57. Sexual behaviors were not observed during interactions between early-adolescent mice, but they were observed among late-adolescent mice. Sexual behavior was not included in the composite measure of SI.

Mice tested in the SCPP and SI experiments were also used in the fear conditioning experiments that compared freezing responses of isolate- and socially housed mice (see **Fig. 11**). Mice tested for SCPP and SI during early adolescence remained in social housing until the beginning of the fear conditioning experiments, while mice that were tested during late adolescence were socially housed after SI testing for 1–5 days before the beginning of the fear conditioning experiments. Mice that were tested for SCPP and SI during early and late adolescence were pseudo-randomized and counterbalanced relative to their social housing condition during the fear conditioning experiments.

### Statistics

Three-, 4- and 5-factor ANOVAs where used to assess behavioral responses, with the genotype of the subjects (BALB or B6), the sex of the subjects (male or female), the conditioning schedule of the objects (paired CS-UCS, unpaired CS-UCS, UCS only or CS only), test trial (CS only or paired CS-UCS) or housing context (social or isolate) as between-group factors, and trial number as a repeated measure. Various combinations of these factors were used depending on the particular experiment. Post-hoc comparisons were carried out either with planned orthogonal comparisons or univariate F-tests that were controlled for type I error with Bonferroni corrections.
